# An Ab Initio Correction Vector Restricted Active Space Approach to the L-Edge XAS and 2p3d RIXS Spectra of Transition Metal Complexes

**DOI:** 10.1021/acs.jctc.3c00663

**Published:** 2023-10-19

**Authors:** Seunghoon Lee, Huanchen Zhai, Garnet Kin-Lic Chan

**Affiliations:** † Department of Chemistry, Seoul National University, Seoul 151-747, South Korea; ‡ Division of Chemistry and Chemical Engineering, California Institute of Technology, Pasadena, California 91125, United States

## Abstract

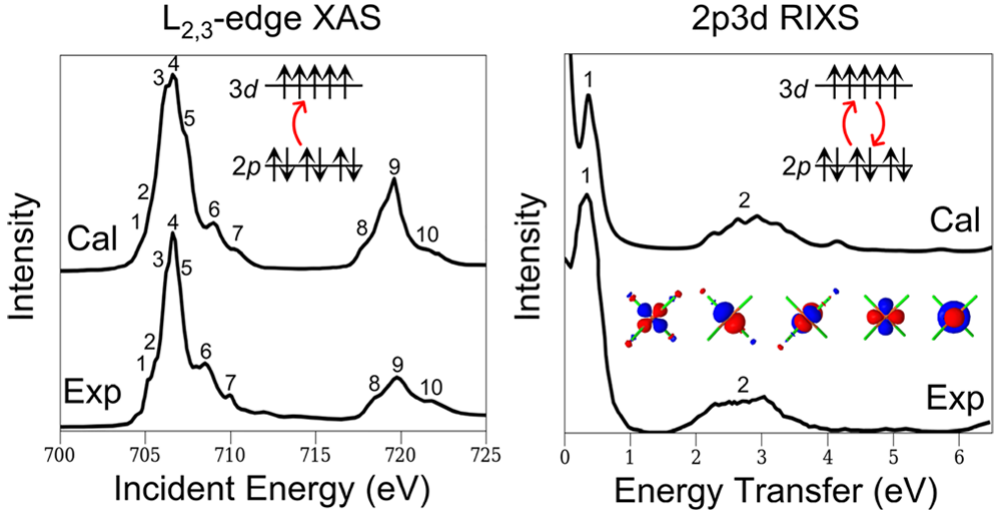

We describe an ab initio approach to simulate L-edge X-ray absorption (XAS) and 2p3d resonant inelastic X-ray scattering (RIXS) spectroscopies. We model the strongly correlated electronic structure within a restricted active space and employ a correction vector formulation instead of sum-over-state expressions for the spectra, thus eliminating the need to calculate a large number of intermediate and final electronic states. We present benchmark simulations of the XAS and RIXS spectra of the iron complexes [FeCl_4_]^1–/2–^ and [Fe(SCH_3_)_4_]^1–/2–^ and interpret the spectra by deconvolving the correction vectors. Our approach represents a step toward simulating the X-ray spectroscopies of larger metal cluster systems that play a pivotal role in biology.

## Introduction

**Figure 1:**
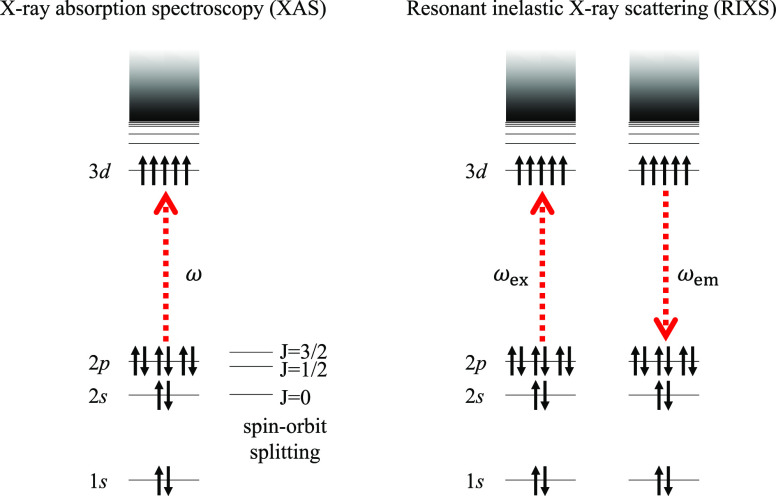
Schematic of the one- and two-step processes of L_2,3_-edge XAS and 2p3d RIXS spectroscopies.

X-ray spectroscopies are indispensable for characterizing the electronic structure of transition metal complexes.^1−4^ For first-row transition metals, L_2,3_-edge X-ray absorption (XAS) and 2p3d resonant inelastic X-ray scattering (RIXS) spectroscopies are of particular interest.^5^ They involve one- and two-step transition processes, respectively, between the 2p core and 3d valence orbitals, as depicted in Figure 1. The dipole-allowed nature of these spectra ensures high intensity and energy resolution. However, the effects of ligand fields, the presence of different multiplet spins, and spin–orbit coupling complicate the interpretation of the spectra. Theoretical models are thus essential.

Recently, several ab initio methods have appeared to compute L-edge XAS^1,4,6^ and RIXS^6^ spectra. These methods use sum-over-state expressions.^1,4,6^ However, such approaches become impractical when there are a large number of intermediate and final states, as is expected to be the case when simulating larger bioinorganic clusters.^2,3^

A route to computing spectra is the correction vector (CV) formulation,^7^ where frequency-dependent response equations are solved to obtain the CVs, which determine the spectrum at each frequency. Here we describe an ab initio implementation of the CV approach for L-edge XAS and 2p3d RIXS spectra within a restricted active space model of the correlated transition metal electronic structure.^8^ The outline of the paper is as follows. In Theoretical Formulation, we introduce the ab initio relativistic Hamiltonian and the CV approach for L-edge XAS and 2p3d RIXS spectra. We also describe how to deconvolve the spectra to separate different electronic effects. In Computational Details, we describe the geometries, molecular orbitals, active space models, and wave function ansatz and methods to optimize the wave function ansatz.^9−11^ In the Results and Discussion, we compute the XAS and RIXS spectra for monomeric ferrous and ferric tetrahedral iron complexes and compare them to available experimental spectra. By deconvolution of the theoretical spectra, we interpret the contributions of different electronic effects and states to the peaks in the XAS and RIXS spectra, highlighting the role of certain electron correlations. In the Conclusion, we provide some perspective on future developments of this approach.

## Theoretical Formulation

### Spin–Orbit Hamiltonian

Simulations of the L_2,3_-edge XAS and 2p3d RIXS spectra both share a process involving the excitation of an electron from the 2p core orbital of the transition metal atom (see Figure 1). As a consequence of relativity, the hole states in the 2p manifold are split. Thus, we start from an ab initio Hamiltonian containing the principal effect of relativity, namely, spin–orbit coupling, described within the mean-field Breit–Pauli (BP) approximation. In second quantization, this is
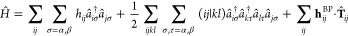
1where *h*_*ij*_ and (*ij*|*kl*) are the ab initio one- and two-electron integrals,
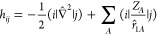
2
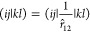
3**h**_*ij*_^BP^ are the mean-field Breit–Pauli matrix elements,
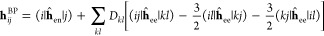
4
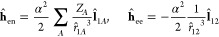
5and **T̂**_*ij*_ (*T̂*_*ij*_^*x*,*y*,*z*^) are the Cartesian triplet operators,
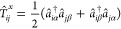
6
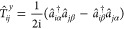
7
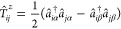
8Further discussion of the Breit–Pauli mean-field Hamiltonian can be found in ref (12).

### XAS/RIXS Spectra

The XAS spectral function (*S*) and RIXS cross section (σ) (averaged over all orientations, directions, and polarizations of the scattered radiation) can be written as
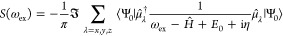
9
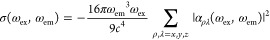
10with
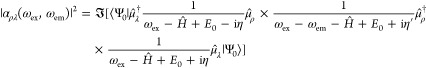
11where |Ψ_0_⟩ and *E*_0_ are the ground-state wave function and energy, **μ̂** is the dipole operator, ω_ex_ and ω_em_ are the energies of the incident and scattered radiation, and η and η′ are Lorentzian broadening factors. The symbol \Im *C* represents the imaginary component of the complex number *C*. We note that we consider only resonant terms in this work.

### Correction Vector Approach

The expressions in eqs 9 and 11 involve resolvent operators *R̂*(*z*) = [*z* – *Ĥ*]^−1^. The correction vector approach to nonlinear properties^7^ involves computing the application of the resolvent to state |*C*⟩ = *R̂*|*X*⟩ by solving (*z* – *Ĥ*)|*C*⟩ = |*X*⟩; |*C*⟩ is termed the correction vector (CV). In this way, the explicit formation of the resolvent is avoided.

Following this, the XAS/RIXS quantities can be computed in three steps: (1) solve for |Ψ_0_⟩ and *E*_0_, (2) solve for the response (from the correction vector equations), and (3) compute *S*/σ from the correction vectors. Specifically, after obtaining |Ψ_0_⟩ and *E*_0_, we compute the CVs ({*A*_λ_(ω_ex_)}) by solving
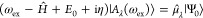
12and obtain the XAS spectral function as
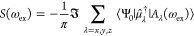
13For the RIXS cross section, we solve for an additional set of CVs ({*B*_*ρλ*_(ω_ex_, ω_em_)}),
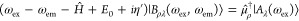
14and compute the cross section using
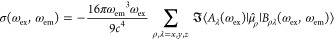
15

### Interpretation of Correction Vectors

The CVs can be formally expanded in a sum over states:
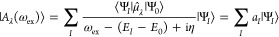
16
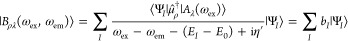
17where Ψ_*I*_ and *E*_*I*_ are eigenstates and eigenvalues of *Ĥ*, respectively. On resonance (i.e., a divergent denominator in eqs 16 and 17), |Ψ_*I*_⟩ is the final state. This has a core-excited character in XAS and valence-excited character in RIXS. Note that final states in XAS are the intermediate states in RIXS.

### Deconvolution

To interpret the L-edge XAS and RIXS spectra, we deconvolve the intermediates into particle–hole and spin contributions. We define the particle–hole components for XAS (*S*_*ia*_^(ph)^(ω_ex_)) and RIXS (σ_*ia*_^(ph)^(ω_ex_, ω_em_)) as
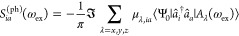
18
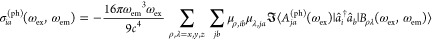
19where the correction vector *A*_*ia*_^(ph)^(ω_ex_) is obtained from
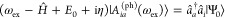
20On resonance, we interpret *S*_*ia*_^(ph)^ as the amplitude of the core (*i*) to valence (*a*) excitation in the XAS final state and σ_*ia*_^(ph)^ as the amplitude of the valence (*i*) to valence (*a*) excitation in the RIXS final state. In XAS, we further define the total valence (particle) contribution by summing over the core (hole) contributions:
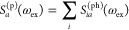
21

To deconvolve the spectra into different spin contributions for XAS (*S*_*S*_^(s)^(ω_ex_)) and RIXS (σ_*S*_^(s)^(ω_ex_, ω_em_)), we apply spin projection operators (*P*_*S*_):
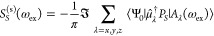
22
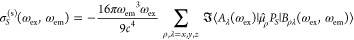
23We use here Löwdin’s spin projector:^13^
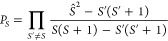
24

For all deconvolution schemes, the sum of the deconvolved spectra is the total spectrum:
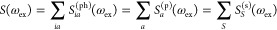
25
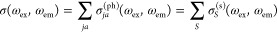
26

## Computational Details

We take the geometries of [FeCl_4_]^1–/2–^ and [Fe(SCH_3_)_4_]^1–/2–^ from previous computational studies.^4,14^ The [Fe(SCH_3_)_4_]^1–^ geometry comes from the X-ray crystal structure, and the geometries of other complexes correspond to the optimized DFT structures. We used the restricted active space (RAS) ansatz for the ground-state wave function (Ψ_0_) and the correction vectors (*A*_λ_ and *B*_λ,ρ_) (see Active Space Models). The RAS ansatz is implemented using matrix product state (MPS) techniques (see Matrix Product State Implementation). We construct the active space using the procedure described in Active Space Construction. For the spectra calculations, we used Lorentzian broadening factors of η = 0.3 eV and η′ = 0.1 eV in eqs 9 and 11. We simulated RIXS spectra using the incident radiation energy (ω_ex_) that results in the maximum intensity of the L_3_ band in the XAS spectra. All calculations were performed using the PySCF^15−17^ and Block2^18^ packages. Simple example scripts for this work are provided in the PyXray GitHub repository.^19^

### Active Space Models

A RAS ansatz can be written in occupation number form as
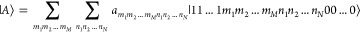
27where *m*_*i*_ and *n*_*j*_ are the occupations (0, 1) in the two active subspaces RAS1 and RAS2, respectively, and *a*_*m*_1_*m*_2_...*m*_*M*_*n*_1_*n*_2_...*n*_*N*__ is the coefficient of the determinant |1 1 ... 1 *m*_1_*m*_2_ ... *m*_*M*_*n*_1_*n*_2_ ... *n*_*N*_ 0 0 ... 0⟩. RAS1 consists of *M* occupied orbitals with a maximum number of holes (*M*_hole_), i.e., ∑_*i*=1_^*M*^*m*_*i*_ ≥ *M* – *M*_hole_. RAS2 consists of *N* orbitals with no restrictions on the electron occupancy, except for the total number of electrons in the RAS spaces, i.e., ∑_*i*=1_^*M*^*m*_*i*_ + ∑_*i*=1_^*N*^*n*_*i*_ = *N*_elec_^RAS^. Additional RAS partitions can be introduced.

For the XAS spectra, we used minimal RAS models with five 3d valence orbitals of Fe in RAS2 and three 2p core orbitals of Fe in RAS1, with *M*_hole_ = 1, which we designate as RAS1(2p_Fe_^6^)RAS2(3d_Fe_^5/6^). For the RIXS spectra, we also considered larger active space models with an additional RAS1 (RAS1′) partition consisting of four σ-bonding orbitals of the Fe–Cl or Fe–S bonds; both RAS1 and RAS1′ have *M*_hole_ = 1. We designate this ansatz RAS1(2p_Fe_^6^)RAS1′(σ_Fe–Cl/S_^8^)RAS2(3d_Fe_^5/6^).

### Matrix Product State Implementation

We implement the RAS ansatz within the MPS formalism. We rewrite the configuration coefficients in eq 27 as
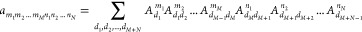
28where *d*_*i*_ is a bond index of dimension *D*, **A**^*m*_*k*_^ (1 < *k* ≤ *M*) and **A**^*n*_*k*_^ (1 ≤ *k* < *N*) are *D* × *D* matrices, and **A**^*m*_1_^ and **A**^*n*_*N*_^ are 1 × *D* and *D* × 1 vectors, respectively. All of the matrices and vectors contain complex elements. Here we choose the bond dimension *D* so that the RAS ansatz is exactly represented: there is no MPS compression, and the MPS formalism is only used to simplify the implementation. The advantages of this implementation arising from using MPS compression compared to the traditional determinant-based approach will be explored in future studies on more complex systems.

To compute ground states, we used the density matrix renormalization group (DMRG) algorithm,^9,20,21^ and we used the dynamical DMRG algorithm^10^ to solve the correction vector equations in eqs 12 and 14.

### Active Space Construction

To construct the active space for the RAS ansatz, we used a technique introduced in a previous study of iron–sulfur clusters.^22^ We utilized the ANO-RCC-VDZP basis set^23^ for all calculations, specifically designed to capture scalar relativistic effects. We first performed unrestricted DFT calculations for the high-spin state without spin–orbit coupling using the BP86 functional.^24,25^ Then we computed unrestricted natural orbitals as the eigenvectors of the sum of α and β DFT density matrices. We then identified active space orbitals from the unrestricted natural orbital occupation numbers and localized the orbitals within the active space to improve the convergence of the DMRG and dynamic DMRG algorithms. Using the active space, we constructed the RAS Hamiltonians from the total Hamiltonian in eq 1 and the RAS ansatz based on the localized natural orbitals in eq 27. Example scripts for the active space construction are available through a software repository.^19^

It is worth noting that this is merely one way to obtain the active space, and the problem of minimal active space construction for X-ray spectroscopy requires a more systematic investigation in general.

## Results and Discussion

Using the above formalism, we note that it is important to highlight that this is merely one approach to constructing the active space model. Determining the specific orbitals to incorporate into the active space in order to achieve enhanced spectra is of paramount importance. This topic will undoubtedly necessitate more rigorous and systematic exploration in upcoming research. We calculated the L-edge XAS and 2p3d RIXS spectra of the [Fe^II/III^Cl_4_]^2–/1–^ and [Fe^II/III^(SCH_3_)_4_]^2–/1–^ complexes. We compare our results to experimental spectra, normalizing the maximum intensity of the L_3_-edge band of XAS to 1 and that of the highest-intensity band of RIXS to 0.2 (for the ferrous complexes), 0.8 (for the tetrachloride ferric complex), and 0.6 (for the tetrathiolate ferric complex). The experimental data are taken from refs (2) and (4) for XAS and ref (3) for RIXS. Note that for the tetrathiolate ferrous and ferric complexes, we used experimental spectra of complexes with benzenethiolate (SPh) and 2,3,5,6-tetramethylbenzenethiolate (SDur) ligands rather than the methylthiolate ligands used in our computations.

### L-Edge XAS of [Fe^II^Cl_4_]^2–^ and [Fe^II^(SCH_3_)_4_]^2–^

**Figure 2:**
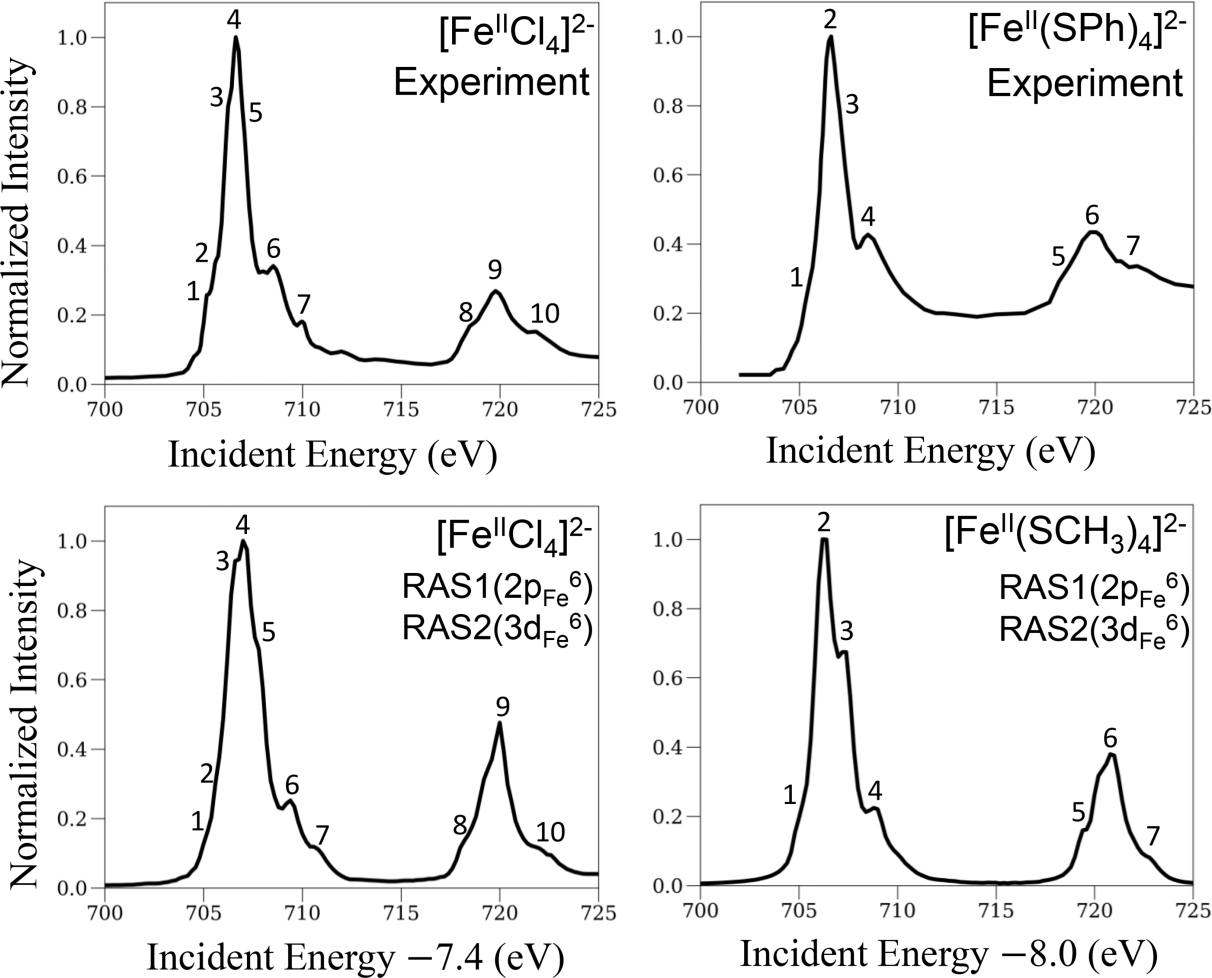
L-edge XAS spectra of ferrous tetrachloride and tetrathiolate complexes (left and right panels, respectively). The experimental spectra in the upper panels are taken from refs (2) and (4). Important features of the experimental spectra are enumerated based on earlier experimental studies,^2,4^ and the corresponding features in the theoretical spectra are enumerated.

We first discuss the simulations of the L-edge XAS spectra for the ferrous complexes [Fe^II^Cl_4_]^2–^ and [Fe^II^(SCH_3_)_4_]^2–^ using the RAS1(2p_Fe_^6^)RAS2(3d_Fe_^6^) active space model. Figure 2 shows the experimental (upper panels) and calculated (lower panels) spectra of [Fe^II^Cl_4_]^2–^ (left panels) and [Fe^II^(SCH_3_)_4_]^2–^ (right panels). The theoretical spectra with the minimal RAS model are in good agreement with the experimental spectra, capturing the relative intensities and relative energy positions of the bands. However, the theoretical spectra for [Fe^II^Cl_4_]^2–^ and [Fe^II^(SCH_3_)_4_]^2–^ are shifted by 7.4 and 8.0 eV, respectively, compared to the experimental spectra (∼10% error). Previous studies using the MRCI method with a single excitation amplitude^4^ also reported a constant energy-shift error of size 8, 3, and 2.7 eV as the size of the cc-pwCVXZ basis set (X) was increased from D to T to Q, while other studies have shown that orbital relaxation also reduces the constant energy-shift error.^26^ Thus, we attribute our constant energy-shift error to the small size and lack of orbital relaxation in the RAS model.

**Figure 3:**
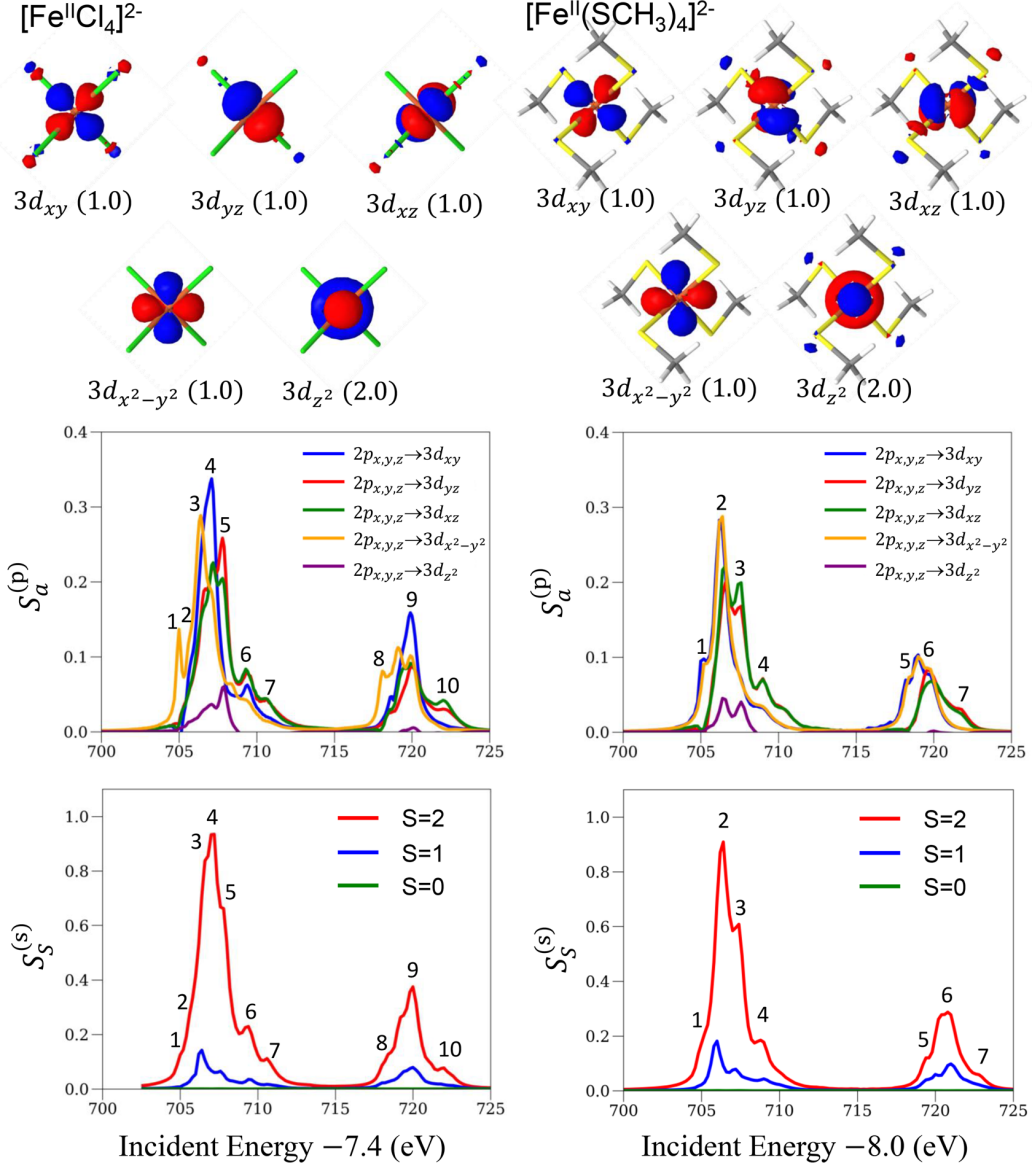
Natural orbitals of the ground-state RAS wave function (top panels) and deconvolved XAS spectra for particle (middle panels) and spin (bottom panels) contributions for [Fe^II^Cl_4_]^2–^ (left panels) and [Fe^II^(SCH_3_)_4_]^2–^ (right panels). The particle contributions are measured in the natural orbital basis shown in the top panels.

We show deconvolved spectra for [Fe^II^Cl_4_]^2–^ and [Fe^II^(SCH_3_)_4_]^2–^ in the left and right panels in Figure 3, respectively. The particle–hole deconvolution was done in the natural orbital basis of the ground-state RAS wave function, and the valence natural orbitals (labeled by atomic orbital character) are shown in the top panels of Figure 3. The value in parentheses for each natural orbital denotes the natural occupation number. In the middle panels of Figure 3, we present the valence (particle) contribution, as defined by summing over the core (hole) indices in the core-to-valence decomposition in eq 21. Each representative band is observed to result from a different valence (particle) contribution, and based on their band positions, we can conclude that the approximate orbital energy order is 3d_*z*^2^_ < 3d_*x*^2^–*y*^2^_ < 3d_*xy*_ < 3d_*yz*_ = 3d_*xz*_ for [Fe^II^Cl_4_]^2–^ and 3d_*z*^2^_ < 3d_*x*^2^–*y*^2^_ = 3d_*xy*_ < 3d_*yz*_ = 3d_*xz*_ for [Fe^II^(SCH_3_)_4_]^2–^.

In the bottom panels of Figure 3, we present the deconvolved spectra for the different spin components. For both [Fe^III^Cl_4_]^1–^ and [Fe^III^(SCH_3_)_4_]^1–^, the largest contribution is for the same spin component as the (high-spin) ground state (*S* = 2), with contributions of 88% and 83%, respectively. The contributions of the Δ*S* = 1 transitions are 12% and 17%, while those of the Δ*S* = 2 transitions are negligible. Interestingly, the percentages of the Δ*S* = 1 contribution are similar for the two complexes, possibly due to similar spin–orbit coupling strengths resulting from the same oxidation state.

### L-Edge XAS of [Fe^III^Cl_4_]^1–^ and [Fe^III^(SCH_3_)_4_]^1–^

**Figure 4:**
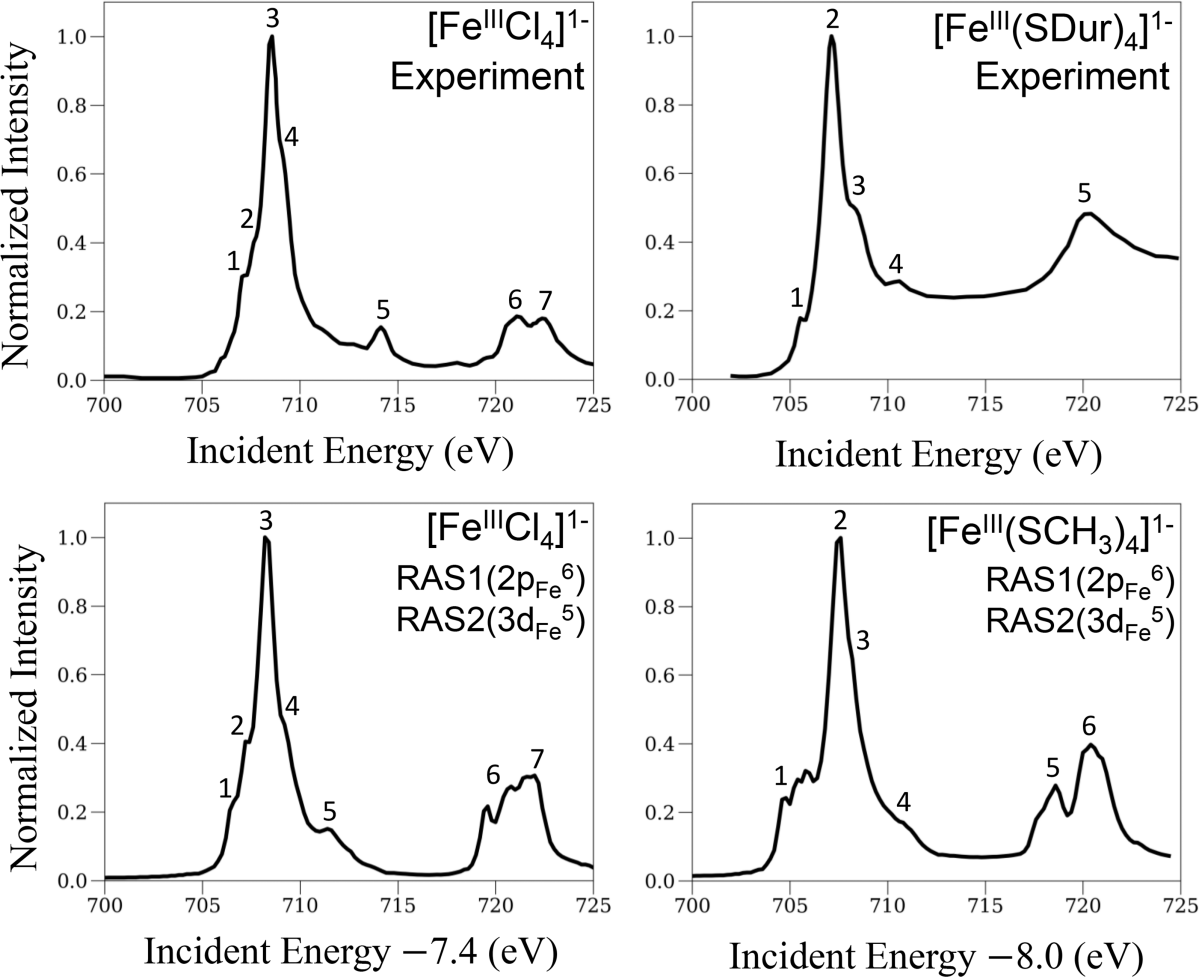
L-edge XAS spectra of ferric tetrachloride and tetrathiolate complexes (left and right panels, respectively) with the same format as in Figure 2.

**Figure 5:**
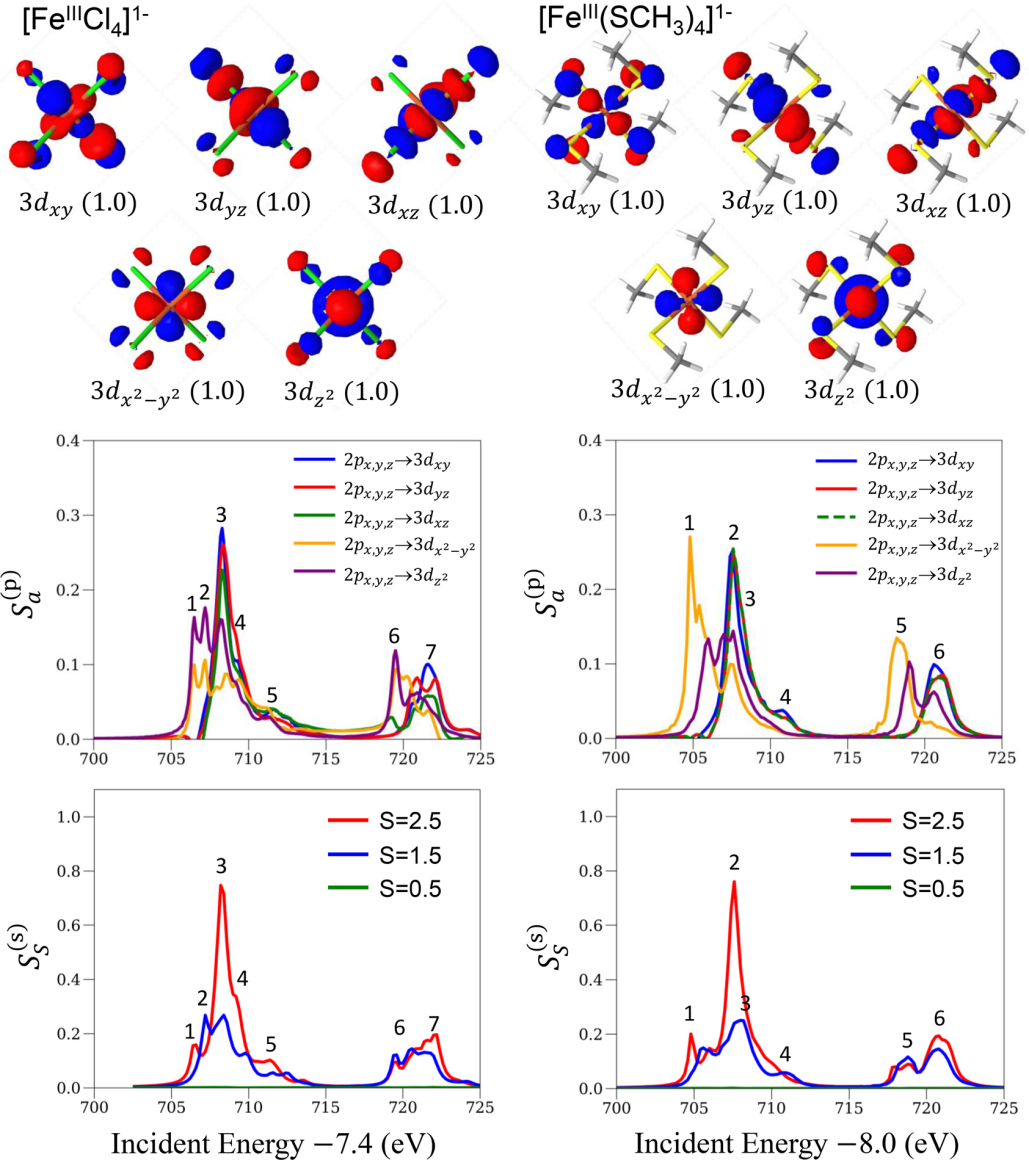
Natural orbitals of the ground-state RAS wave function (top panels) and deconvolved XAS spectra for particle (middle panels) and spin contributions (bottom panels) for [Fe^III^Cl_4_]^1–^ (left panels) and [Fe^III^(SCH_3_)_4_]^1–^ (right panels). The particle contributions are measured in the natural orbital basis shown in the top panels.

We next present XAS spectra of the ferric complexes in Figure 4. The theoretical spectra of [Fe^III^Cl_4_]^1–^ and [Fe^III^(SCH_3_)_4_]^1–^ have similar constant energy-shift errors of 7.4 and 8.0 eV, respectively. The general features of the theoretical XAS spectra are in good agreement with the experimental ones. However, the relative positions of some bands do not match up, unlike in the ferrous complexes. For example, in [Fe^III^Cl_4_]^1–^, the energy position of the fifth band is shifted relative to the highest peak by 2.5 eV. In an earlier MRCI and MREOM-CC study,^4^ this shift was attributed to multireference electron correlation effects involving the ligand 3p orbitals and metal 3d and 4s orbitals. In [Fe^III^(SCH_3_)_4_]^1–^, in the theoretical spectrum, the first band has a broader shoulder, the third band is closer to the second band, and the L_2_ edge has two clear bands, as opposed to only one in the experiment. These differences can be better understood using the deconvolved spectra in Figure 5, which we now discuss.

The top panels of Figure 5 represent the valence orbitals of the natural orbitals for the ground-state RAS wave functions. In contrast to the ferrous complexes, the natural orbitals of the ferric complexes have significant mixing with 3p orbitals of the ligands due to the higher oxidation state of Fe. An exception to this is the 3d_*x*^2^–*y*^2^_ orbital of [Fe^III^(SCH_3_)_4_]^1–^, which has little mixing. The middle panels of Figure 5 show the particle (valence) contributions. In [Fe^III^Cl_4_]^1–^ and [Fe^III^(SCH_3_)_4_]^1–^, the band positions of 3d_*xy*_, 3d_*yz*_, and 3d_*xz*_ are identical. In [Fe^III^Cl_4_]^1–^, the band positions of 3d_*x*^2^–*y*^2^_ and 3d_*z*^2^_ are also the same. Thus, the approximate orbital energy order is 3d_*z*^2^_ = 3d_*x*^2^–*y*^2^_ < 3d_*xy*_ = 3d_*yz*_ = 3d_*xz*_, in agreement with an ideal tetrahedral ligand. However, in [Fe^III^(SCH_3_)_4_]^1–^, the band positions of 3d_*x*^2^–*y*^2^_ are shifted by −2 eV relative to those of 3d_*z*^2^_. The shift in the 3d_*x*^2^–*y*^2^_ bands is the main reason for the disagreement between the theoretical and experimental spectra in the right panels of Figure 4. The small mixing between the 3d_*x*^2^–*y*^2^_ orbital and the 3p ligand orbitals in Kohn–Sham density functional theory, used to construct the active space, contributes to this shift.

In the bottom panels, we present the deconvolved spectra for the different spin components. For both complexes, the largest contribution (60%) comes from states with the same spin as the (high-spin) ground states (*S* = 2.5). Contributions of 40% are observed for the Δ*S* = 1 transition for both ferric complexes, while the contribution of the Δ*S* = 2 transition is negligible. The higher contributions for the Δ*S* = 1 transition in the ferric complexes compared to the ferrous complexes can be attributed to the stronger spin–orbit coupling due to the higher oxidation state of Fe.

### RIXS Spectra of [Fe^II^Cl_4_]^2–^ and [Fe^II^(SCH_3_)_4_]^2–^

**Figure 6:**
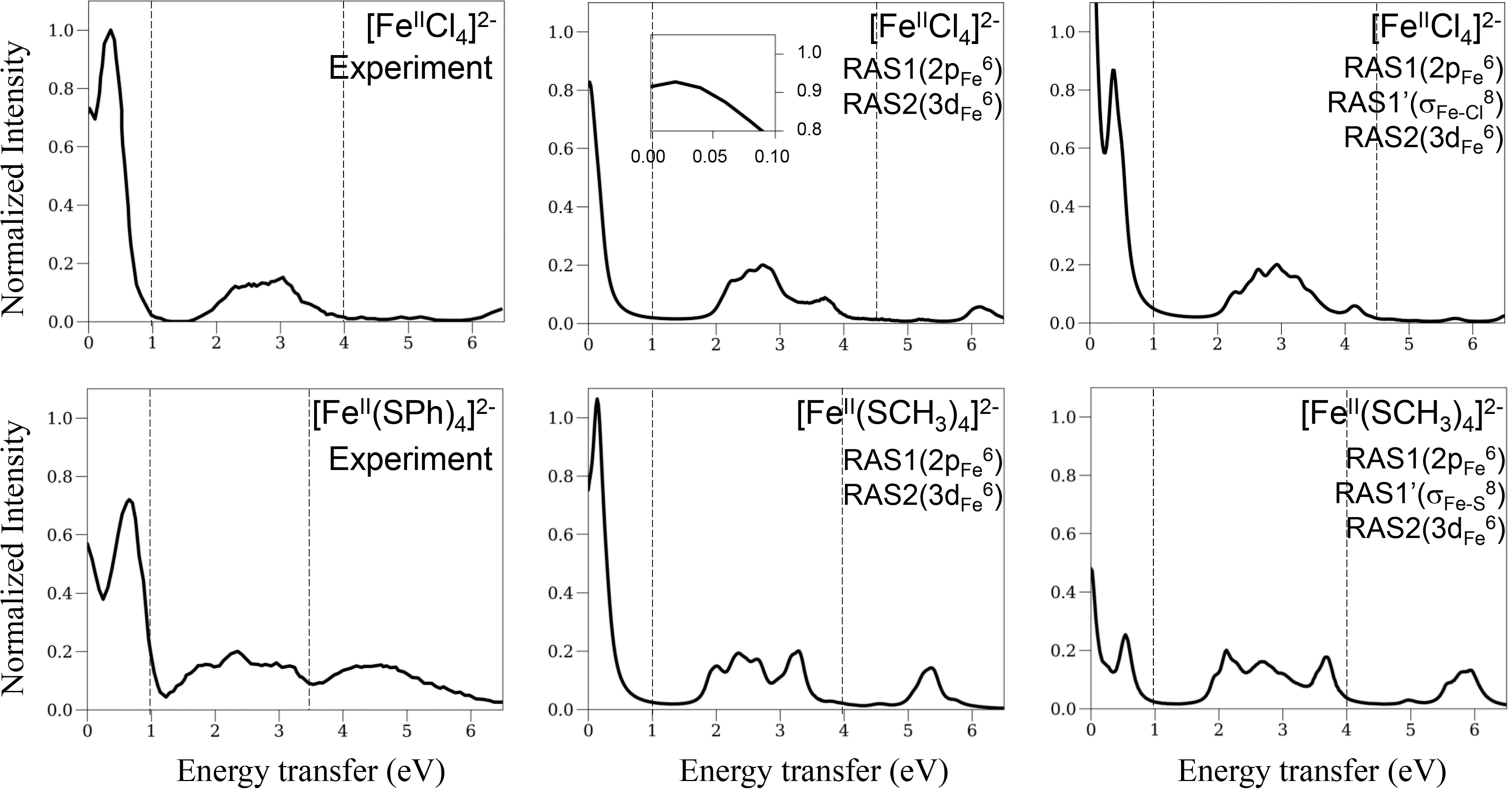
2p3d RIXS spectra of [Fe^II^Cl_4_]^2–^ and [Fe^II^(SCH_3_)_4_]^2–^ (top and bottom panels, respectively). The experimental spectra in the left panels are from ref (3). Theoretical spectra with two different active space models are shown in the center and right panels.

Next, we discuss the 2p3d RIXS spectra of the ferrous complexes [Fe^II^Cl_4_]^2–^ and [Fe^II^(SCH_3_)_4_]^2–^. Figure 6 shows the experimental spectra (left panels) and theoretical spectra (center and right panels) with different active space models (see Computational Details). For convenience, we divide the spectra into three regions divided by the black dashed lines. In the first region (0–1 eV), there are two bands at 0.00 and 0.35 eV for [Fe^II^Cl_4_]^2–^ and at 0.00 and 0.67 eV for [Fe^II^(SCH_3_)_4_]^2–^ in the experimental spectra. However, we observed only one band in the theoretical spectra using the minimal active space at 0.02 eV for [Fe^II^Cl_4_]^2–^ and 0.14 eV for [Fe^II^(SCH_3_)_4_]^2–^. When we change to the larger RAS1(2p_Fe_^6^)RAS1′(σ_Fe–Cl/S_^8^)RAS2(3d_Fe_^5/6^) active space (that includes the four occupied σ-bonding orbitals between the Fe and Cl/S atoms), two bands correctly appear, at 0.00 and 0.36 eV for [Fe^II^Cl_4_]^2–^ and 0.00 and 0.54 eV for [Fe^II^(SCH_3_)_4_]^2–^. This emphasizes the importance of electron correlation between the 3d orbitals and the σ-bonding orbitals to reproducing the energy splitting in this energy range. (We expect that the same correlation would shift the bands of the XAS spectra in Figure 2 by approximately 0.3 eV, but as these peaks correspond to different d orbital states (and not charge-transfer excitations), we do not expect a qualitative change in the features of the spectra).

In the second energy region (1–4 eV), the experimental spectra contain broad bands, with half-maximum intensity (HM) at 2.10 and 3.31 eV for the chloride complex and 1.49 and 3.45 eV for the thiolate complex and with full widths at half-maximum (fwhm) of 1.21 and 1.96 eV, respectively. The minimal active space model has narrower bands with HM at 2.12 and 3.07 eV (chloride complex) and 1.86 and 3.42 eV (thiolate complex) and fwhm of 0.95 and 1.56 eV. Including the σ-bonding orbitals broadens the bands with HM at 2.24 and 3.50 eV (chloride complex) and 1.88 and 3.84 eV (thiolate complex) and fwhm of 1.26 and 1.96 eV. These latter results are closer to what is seen in experiment but are slightly shifted to positive energy.

In the third region of the experimental spectra (4 to 6 eV), there is no representative band for the chloride complex, while there is a broad band in the range of 3.5 to 6 eV for the thiolate complex. The corresponding band in the theoretical spectra for the thiolate complex is much narrower than the experimental band. We ascribe the difference to missing certain important states, such as ligand-to-metal charge transfer (LMCT) states, in the active space models.

**Figure 7:**
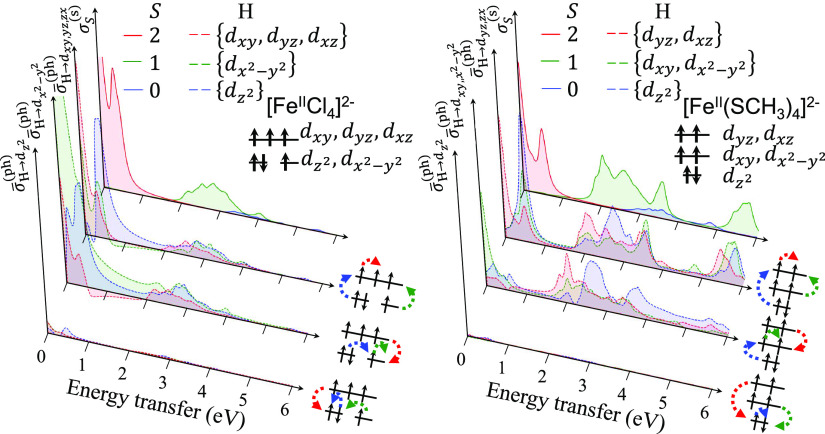
Deconvolved theoretical RIXS spectra for [Fe^II^Cl_4_]^2–^ and [Fe^II^(SCH_3_)_4_]^2–^ complexes (left and right panels, respectively) using the larger active space model. Each panel shows four deconvolved spectra. From front to back, the first three show the average particle–hole (valence-to-valence excitation) contributions (σ̅_H→P_^(ph)^ in eq 29) for three particle sets (P) (scale is in arbitrary units). In each spectrum, the different contributions of the three hole sets (H) are represented by dashed lines of different colors, and the corresponding transitions are depicted in the orbital diagram next to the *x* axis by dashed arrows (using the same color scheme) next to the *x* axis. The inset depicts the approximate ground-state electronic configuration and the three sets of 3d orbitals used to compute the average particle–hole contributions. The remaining graph at the rear represents the spin-state contribution to the final states (σ_*S*_^(s)^) defined in eq 23.

We further analyzed the RIXS spectra of the ferrous complexes (in the larger active space model) by deconvolving them, as shown in Figure 7. In each panel, the spectrum in the rearmost position shows the spin-state deconvolution defined in eq 23. The ground state for both complexes is a spin quintet (*S* = 2). Thus, the red curve, which depicts quintet contributions, shows the spin-allowed transitions, while the green and blue curves depict spin-forbidden transitions with Δ*S* = 1 and 2, respectively. The remaining three spectra from front to back represent the average particle–hole contributions defined as follows. (To simplify the analysis, we considered only valence-to-valence transitions, which account for over 99% of the total spectrum). In addition, we partition the five 3d orbitals into three sets: {d_*z*^2^_}, {d_*x*^2^–*y*^2^_}, {d_*xy*_, d_*yz*_, d_*xz*_} for [Fe^II^Cl_4_]^2–^ and {d_*z*^2^_}, {d_*x*^2^–*y*^2^_, d_*xy*_}, {d_*yz*_, d_*xz*_} for [Fe^II^(SCH_3_)_4_]^2–^. The average particle–hole contribution is defined by
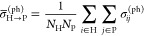
29where *N*_H_ and *N*_P_ are the number of orbitals in the H and P sets and σ_*ij*_^(ph)^ is defined in eq 19. For example,  = 1 and *N*_d_*xy*,*yz*,*zx*__ = 3 for [Fe^II^Cl_4_]^2–^, giving  = .

The deconvolved spectra for the [Fe^II^Cl_4_]^2–^ complex are shown in the left panel of Figure 7. Throughout the energy range of 0–6 eV, the main contributions come from transitions to d_*x*^2^–*y*^2^_ and d_*xy*,*yz*,*xz*_. The two dominant bands in the 0–1 eV range originate from spin-allowed transitions of d_*z*^2^_ → d_*x*^2^–*y*^2^_ and d_*z*^2^_ → d_*xy*,*yz*,*xz*_. We also observe minor contributions from other transitions, which we attribute to the multireference nature of the quintet excited states, as they cannot be described by a single electronic configuration. The broad band in the energy range 1–4 eV primarily results from spin-forbidden transitions with Δ*S* = 1. The spin-flip transitions of d_*xy*,*yz*,*xz*_ → d_*xy*,*yz*,*xz*_, d_*x*^2^–*y*^2^_ and d_*x*^2^–*y*^2^_ → d_*x*^2^–*y*^2^_ contribute to the lower-energy region of the broad band, while the spin-flip transitions of d_*x*^2^–*y*^2^_ → d_*xy*,*yz*,*xz*_, d_*x*^2^–*y*^2^_ and d_*z*^2^_ → d_*xy*,*yz*,*xz*_, d_*x*^2^–*y*^2^_ contribute to the higher-energy region of the band. The Δ*S* = 2 contribution suggests the presence of double spin-flip excitations.

The right panel of Figure 7 shows the deconvolved spectra for the [Fe^II^(SCH_3_)_4_]^2–^ complex. As with the chloride complex, the main transitions are to d_*x*^2^–*y*^2^_ and d_*xy*,*yz*,*xz*_. The bands in the 0–1 eV range are primarily due to spin-allowed transitions of d_*z*^2^_ → d_*yz*,*xz*_ (maximum intensity at 0.54 eV) and minor transitions of d_*z*^2^_ → d_*xy*,*x*^2^–*y*^2^_ (maximum intensity at 0.20 eV). Like the chloride complex, spin-forbidden transitions of Δ*S* = 1 mainly contribute to the broad band in the 1–4 eV range, with a minor contribution from Δ*S* = 2 transitions in the 3–4 eV range. The band can be further divided into three parts based on the dominant spin-flip transitions: transitions of d_*yz*,*xz*_ → d_*xy*,*x*^2^–*y*^2^_, d_*yz*,*zx*_ and d_*xy*,*x*^2^–*y*^2^_ → d_*xy*,*x*^2^–*y*^2^_, d_*yz*,*zx*_, transitions of d_*z*^2^_ → d_*xy*,*x*^2^–*y*^2^_, d_*yz*,*zx*_, and transitions of d_*x*^2^–*y*^2^,*xy*_, d_*yz*,*xz*_, d_*z*^2^_ → d_*yz*,*xz*_ and d_*z*^2^_ → d_*xy*,*x*^2^–*y*^2^_. In the energy range of 4–6 eV, the d–d contributions are similar to those in the higher part of the broad band in the 3–4 eV range.

### RIXS Spectra of [Fe^III^Cl_4_]^1–^ and [Fe^III^(SCH_3_)_4_]^1–^

**Figure 8:**
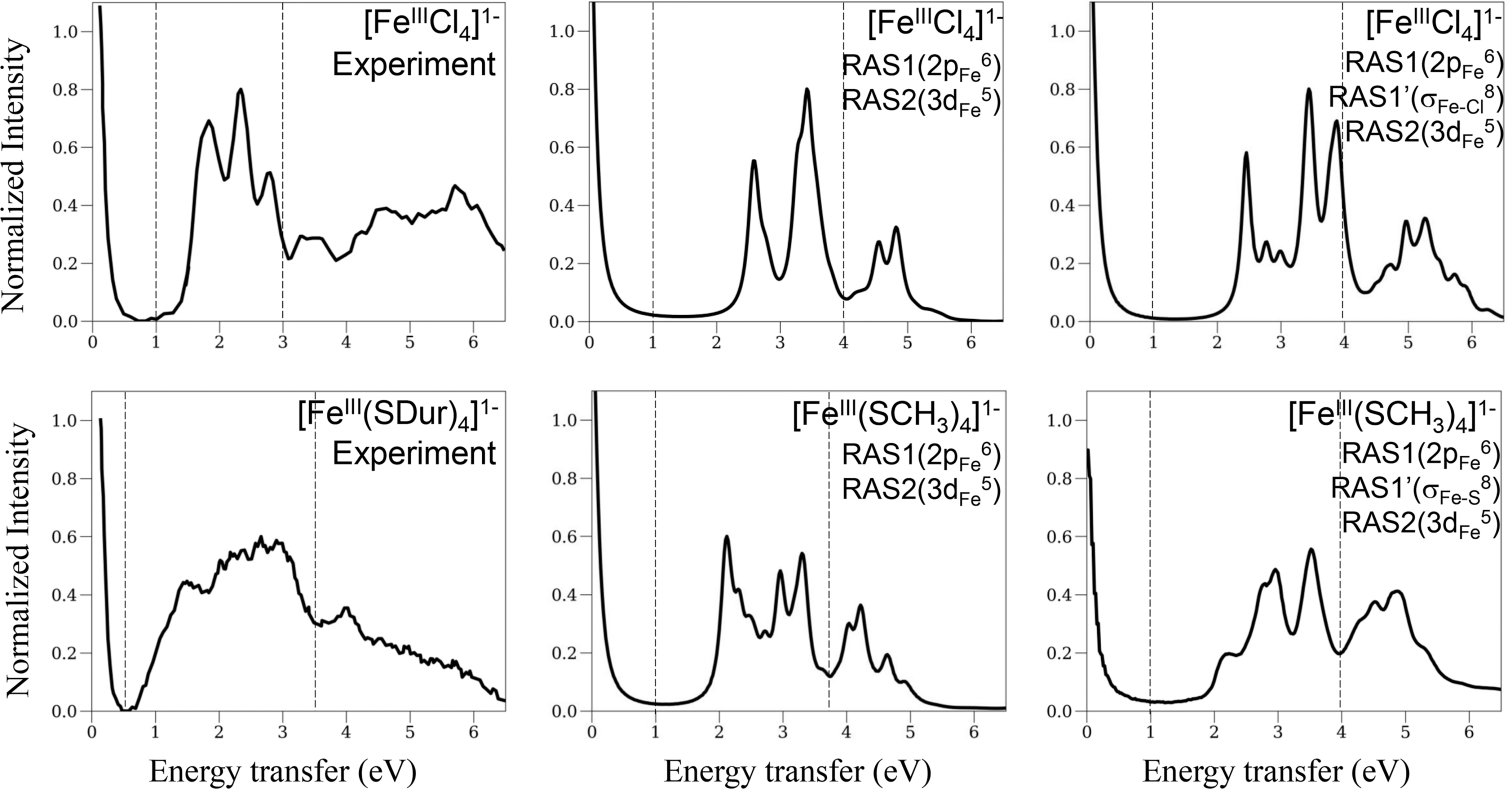
2p3d RIXS spectra of [Fe^III^Cl_4_]^1–^ and [Fe^III^(SCH_3_)_4_]^1–^ (upper and lower panels, respectively), with the same format as in Figure 6.

Similarly, we divide the RIXS spectra of the ferric complexes into three regions, as shown in Figure 8. In the first region (0–1 eV), both the experimental (left panels) and theoretical spectra with different active space models (center and right panels) show a single band at 0 eV, as in the experiment. In the second region (1–4 eV), the experimental spectrum of the chloride complex shows three representative bands at 1.83, 2.34, and 2.80 eV, while the thiolate complex exhibits a broader band with indistinct peaks in the range of 0.5–3.5 eV. The minimal active space model yields two main bands at 2.58 and 3.42 eV for the chloride complex and a slightly broader band in the range of 2–3.5 eV for the thiolate complex. In the chloride complex, the larger active space model splits the two bands into three main bands at 2.46, 3.44, and 3.88 eV and two minor bands at 2.78 and 3.00 eV. In the thiolate complex, in the larger active space the bands away from 0 eV are broadened but still do not match the widths of the experimental bands. Experimental measurements using magnetic circular dichroism (MCD) spectroscopy^27^ show d–d transition bands between 0.9–1.39 eV, while the LMCT bands begin at ∼1.6 eV and extend to ∼3.7 eV for [Fe^III^(SDur)_4_]^1–^. It is likely that the missing LMCT contributions in the theoretical active space lead to bands that are too narrow and are positively shifted as well as the absence of bands and features in the high-energy region (>4 eV).

**Figure 9:**
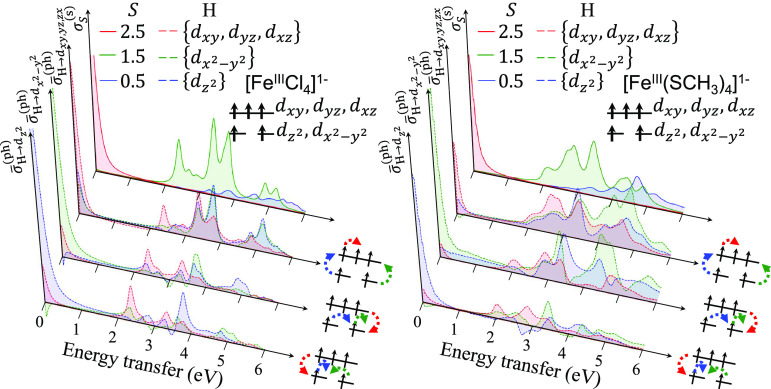
Deconvolved theoretical RIXS spectra for [Fe^III^Cl_4_]^1–^ and [Fe^III^(SCH_3_)_4_]^1–^ (left and right panels, respectively), with the same format as in Figure 7.

Finally, we deconvolve the theoretical spectra of the larger active space model using the same schemes as used in Figure 7. Figure 9 shows the deconvolved spectra for [Fe^III^Cl_4_]^1–^ and [Fe^III^(SCH_3_)_4_]^1–^ in the left and right panels, respectively. As in the ferrous complex, the first region (0–1 eV) is dominated by spin-allowed transitions, while the second and third regions (1–6 eV) are characterized by spin-forbidden transitions of Δ*S* = 1, 2. We observe that in the ferric complexes there is a larger contribution from the Δ*S* = 2 transitions compared to the ferrous complexes. Furthermore, the other spectra in the front and middle panels show the average particle–hole contributions from three sets of particles and holes, namely, {d_*z*^2^_}, {d_*x*^2^–*y*^2^_}, and {d_*xy*_, d_*yz*_, d_*xz*_}. In contrast to the ferrous complexes, all transitions to d_*z*^2^_, d_*x*^2^–*y*^2^_, and d_*xy*,*yz*,*xz*_ contribute to the total spectra across the entire energy range of 0–6 eV in the ferric complexes. The low-energy part of the band in the 2–3 eV region is dominated by spin-flip transitions of d_*xy*,*yz*,*xz*_ → d_*z*^2^_, d_*x*^2^–*y*^2^_, and d_*xy*,*yz*,*xz*_ character, while above this window there is a mixture of various particle–hole contributions.

## Conclusion

In this work, we presented a new ab initio technique to compute L_2,3_-edge XAS and 2p3d RIXS spectra based on the correction vector approach and a restricted active space ansatz. We obtain good general agreement between our theoretical simulations and experimental spectra in a set of mononuclear tetrahedral ferrous and ferric iron complexes. Our results highlight the importance of selecting an appropriate active space for the spectroscopy and the role of electron correlation between the metal and ligand electrons in determining certain spectral features. Improved simulations should incorporate additional orbitals into the active space treatment, for example, to treat LMCT/MLCT states; improve the active space through orbital optimization; and include dynamic correlation effects in the spectra.

The elimination of a sum-over-states computation in the correction vector formulation of XAS and RIXS spectra removes a major limitation in the simulation of spectra for multinuclear transition metal complexes. Simulations of larger iron–sulfur cluster X-ray spectra are currently underway in our group and will be presented elsewhere.

## Supporting Information Available

Table of XAS and RIXS data (XLSX)
